# GZMK+CD8+ T cells Target A Specific Acinar Cell Type in Sjögren’s Disease

**DOI:** 10.21203/rs.3.rs-3601404/v2

**Published:** 2024-07-11

**Authors:** Thomas J.F. Pranzatelli, Paola Perez, Anson Ku, Bruno Matuck, Khoa Huynh, Shunsuke Sakai, Mehdi Abed, Shyh-Ing Jang, Eiko Yamada, Kalie Dominick, Zara Ahmed, Amanda Oliver, Rachael Wasikowski, Quinn T. Easter, Alan N. Baer, Eileen Pelayo, Zohreh Khavandgar, David E. Kleiner, M. Teresa Magone, Sarthak Gupta, Christopher Lessard, A. Darise Farris, Peter D. Burbelo, Daniel Martin, Robert J. Morell, Changyu Zheng, Nicholas Rachmaninoff, Jose Maldonado-Ortiz, Xufeng Qu, Marit Aure, Mohammad H. Dezfulian, Ross Lake, Sarah Teichmann, Daniel L. Barber, Lam C. Tsoi, Adam G. Sowalsky, Katarzyna M. Tyc, Jinze Liu, Johann Gudjonsson, Kevin M. Byrd, Philip L.F. Johnson, John A. Chiorini, Blake M. Warner

**Affiliations:** 1 Adeno-Associated Virus Biology Section, National Institute of Dental and Craniofacial Research, National Institutes of Health, Bethesda, MD, USA.; 2 Department of Biology, University of Maryland College Park, MD, USA.; 3 Salivary Disorders Unit, National Institute of Dental and Craniofacial Research, National Institutes of Health, Bethesda, MD, USA; 4 Laboratory of Genitourinary Cancer Pathogenesis, Center for Cancer Research, National Cancer Institute, National Institutes of Health, Bethesda, Bethesda, MD, USA.; 5 Lab of Oral & Craniofacial Innovation (LOCI), Department of Innovation & Technology Research, ADA Science & Research Institute, Gaithersburg, MD, USA; 6 Department of Biostatistics, Virginia Commonwealth University, VA, USA.; 7 T-lymphocyte Biology Section, National Institute of Allergy and Infectious Diseases, National Institutes of Health, Bethesda, MD, USA; 8 Wellcome Sanger Institute, Wellcome Genome Campus, Hinxton, Cambridge, UK; 9 Department of Dermatology, Michigan Medicine, University of Michigan, Ann Arbor, MI, USA; 10 Sjögren’s Clinical Investigations Team, National Institute of Dental and Craniofacial Research, National Institutes of Health, Bethesda, MD, USA; 11 Laboratory of Pathology, Center for Cancer Research, National Cancer Institute, National Institutes of Health, Bethesda MD, USA.; 12 Consult Services Section, National Eye Institute, National Institutes of Health, Bethesda MD, USA.; 13 Lupus Clinical Trials Unit, National Institute of Arthritis and Musculoskeletal and Skin, Diseases, National Institutes of Health, Bethesda MD, USA.; 14 Genes & Human Disease Research Program, Oklahoma Medical Research Foundation, Oklahoma City, OK, USA.; 15 Arthritis & Clinical Immunology Research Program, Oklahoma Medical Research Foundation, Oklahoma City, OK, USA.; 16 Genomics and Computational Biology Core, National Institute on Deafness and Other Communication Disorders, National Institutes of Health, Bethesda, MD, USA.; 17 Massey Cancer Center, Virginia Commonwealth University, Richmond, VA, USA.; 18 Matrix and Morphogenesis Section, National Institute of Dental and Craniofacial Research, National Institutes of Health, Bethesda, MD, USA; 19 Department of Genetics, Harvard Medical School, Boston, MA, USA.; 20 Laboratory of Genitourinary Cancer Pathogenesis (LCGP) Microscopy Core Facility, Center for Cancer Research, National Cancer Institute, National Institutes of Health, Bethesda, Bethesda, MD, USA; 21 Theory of Condensed Matter, Cavendish Laboratory/Dept Physics, University of Cambridge, JJ Thomson Ave, Cambridge CB3 0HE, UK

## Abstract

Sjögren’s Disease (SjD) is a systemic autoimmune disease without a clear etiology or effective therapy. Utilizing unbiased single-cell and spatial transcriptomics to analyze human minor salivary glands in health and disease we developed a comprehensive understanding of the cellular landscape of healthy salivary glands and how that landscape changes in SjD patients. We identified novel seromucous acinar cell types and identified a population of *PRR4+CST3+WFDC2*− seromucous acinar cells that are particularly targeted in SjD. Notably, *GZMK*+CD8 T cells, enriched in SjD, exhibited a cytotoxic phenotype and were physically associated with immune-engaged epithelial cells in disease. These findings shed light on the immune response’s impact on transitioning acinar cells with high levels of secretion and explain the loss of this specific cell population in SjD. This study explores the complex interplay of varied cell types in the salivary glands and their role in the pathology of Sjögren’s Disease.

## INTRODUCTION

Sjögren’s Disease (SjD) is the second most common systemic autoimmune disease (0.1–4% prevalence across studies in different countries) with a striking 9:1 female to male preponderance and a dramatically increased lifetime risk of lymphomagenesis^1,2^. Despite important advances in understanding the pathogenesis of SjD, neither a unifying etiology nor an efficacious medication that meaningfully improves quality of life or preserves organ function has been established. The inherent clinical and biological heterogeneity of SjD is cited as the main factor that has limited the clinical success of identified targets and developed agents^3^. SjD is characterized by chronic lymphocytic infiltration of the exocrine organs and reduced secretory function. The associated clinical sequelae include xerostomia (i.e., dry mouth) and xerophthalmia (i.e., dry eyes). Additional organ systems may be involved as salivary-type exocrine organs support hydration, lubrication, and antimicrobial activity for multiple organ systems and tissues (e.g., upper and lower airway [nasal cavity, trachea, bronchus], genitourinary tract [Bartholin’s, endocervical glands], digestion [pancreas]) exemplifying the systemic nature of the disease. SjD patients also experience debilitating extra-glandular systemic effects including fatigue, widespread musculoskeletal pain, and polyarthritis^4,5^. There are no effective and approved therapies for SjD and management remains suboptimal but includes palliative and supportive care for symptoms and immunosuppressants for more severe and systemic symptoms.

The etiological basis of SjD suggests a complex interaction between genetics, environmental insults, and hormonal factors. Multiple biological pathways, including both the innate and adaptive immune systems, are reported to be involved in SjD. Genome-wide association studies (GWAS)^6,7^ have now identified 22 single nucleotide polymorphisms (SNPs) associated with SjD. Using polygenic risk scores, the genetic risk of SjD divides the SjD population into anti-SSA autoantibody positive patients, with genetic association dominated by human leukocyte antigens (HLA) SNPs, and anti-SSA autoantibody negative patients, whose SNPs are limited in number, have a more muted effect size, and are unrelated to HLA SNPs^6^. How these risk haplotypes translate to cellular function and autoimmune pathogenesis in targeted organs is not entirely clear.

The salivary glands are composed primarily of acinar cells and ducts, with associated stromal tissues and tissue-resident inflammatory cells. Fluid and the majority of protein secretion in the salivary glands initiates in the acinar cells^8^. In humans, there are three sets of major salivary glands: parotid, composed purely of serous acinar cells; submandibular, a mixture of serous/seromucous cells and mucous acinar cells; and sublingual, predominantly mucous cells. In addition to these three pairs of major glands, hundreds to thousands of minor (submucosal) glands are distributed throughout the oral cavity and upper aerodigestive tract. Minor salivary gland (salivary glands) biopsies are used to evaluate the histopathological component of SjD classification criteria and serve as biological surrogates for major gland involvement^9,10^. The SjD-affected salivary glands exhibits a chronic, mixed, predominantly lymphocytic infiltrate that surrounds ducts and interdigitating with acini in areas not exhibiting non-specific damage (i.e., interstitial fibrosis, ductal dilation, and acinar atrophy)^11–13^. The immune cells contributing to this infiltrate are T lymphocytes (CD4 > CD8), B lymphocytes, and plasma cells; other immune cells are also found, although in fewer numbers including natural killer (NK) cells, macrophages, and dendritic cells^10,14,15^. In some patients, tertiary lymphoid-like structures can also be found^16^. The immunopathology of SjD is modulated by both genetics^17,18^ and clinical features of disease (e.g., anti-SSA+^19^, Age^1^). Advances in genetic, epigenetic, transcriptomic, and proteomic technologies have advanced our understanding of the pathogenesis of SjD. These studies have shown that multiple inflammatory cytokines are involved including: Type I and Type II interferons^20,21^, interleukins^20,22^, and chemokines^23–26^. Emerging technologies (e.g., single cell and spatial transcriptomics) are unraveling disease-specific cellular networks and signaling pathways, and spotlight effector cells and therapeutic pathways.

To date, most attention in SjD has been towards understanding the B cell and CD4 T cell involvement in SjD. The direct involvement of CD8 T cells in SjD requires further study. CD8 T cells, also known as cytotoxic T cells, are a subset of T cells involved in the immune response to viral infections, cancer, and autoimmune diseases. In Sjögren’s disease, CD8 T cells are thought to target the acinar and/or ductal epithelial cells in the salivary gland, leading to tissue damage and glandular dysfunction. However, direct evidence of CD8 T cells exerting cytotoxic effects on epithelial cells is lacking, their phenotypic profile is unknown, and the specific targeted epithelial cells (e.g., a specific type of acinar or ductal cell, or another cell type) or the antigens initiating or driving disease have not been reported.

Most of what is understood about the pathogenesis of SjD in the salivary glands is based on unbiased bulk approaches (e.g., microarray, RNAseq) or on low-throughput methods using focused panels of target genes or proteins. There is a critical need for an unbiased understanding of the molecular changes in the salivary glands at the cell level within the tissue context. Herein, we employed a multifaceted approach, combining the strengths of scRNA-seq and spatial transcriptomics, ex vivo cellular assays, and high-throughput microscopy methods to achieve a comprehensive map of the cellular composition and cell-state changes in human salivary glands in SjD patients. We then determine the disease-specific cellular interactions involved in the immune insult in the gland and uncover novel pathways driving the loss of gland function and parenchymal maintenance. This evidence further supports and also sheds light on the pivotal role of T cells in the pathogenesis of SjD, including direct effects on the exocrine epithelia.

## RESULTS

### Single cell and spatial transcriptomics of the minor salivary glands in Sjögren’s Disease.

**Figure 1:**
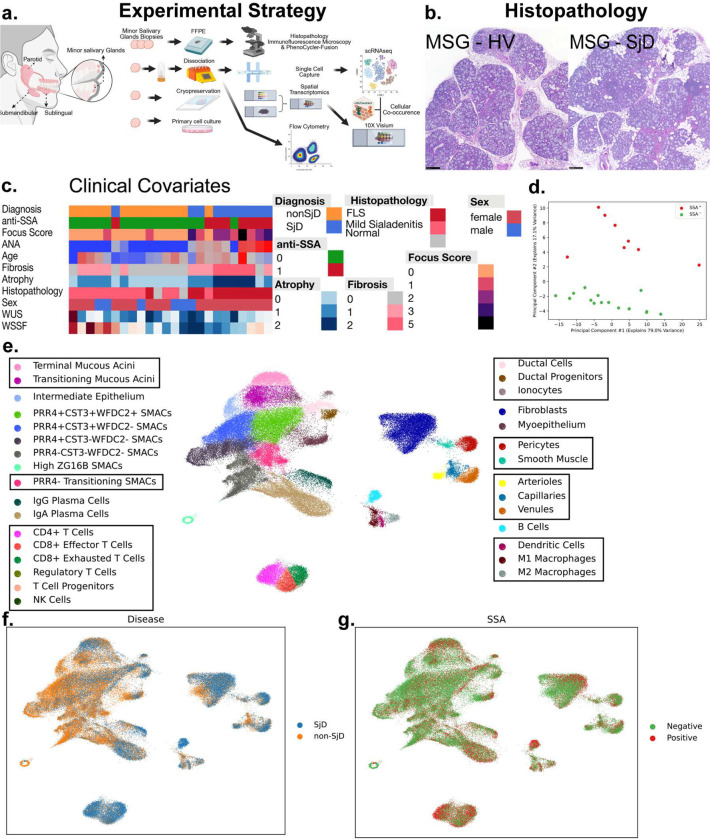
Clinical research investigations for 25 subjects included comprehensive oral, rheumatological, and ophthalmologic investigations applying American College of Rheumatology 2016 Sjögren’s Disease Classification Criteria including salivary gland biopsies on all subjects. Single-cell RNA-sequencing data from 94,227 cells was gathered across 25 patients with and without SjD disease. (a) A cartoon depicting the sample collection from subjects and the creation of scRNA-seq and spRNA-seq libraries. Histological interpretations were rendered on all subjects and patients’ glands. Additional subjects, not included in the scRNAseq analyses, were used for flow cytometry, 10X Visium spatial transcriptomics, multiplex fluorescent in situ hybridization (PhenoCycler-Fusion), and multiplex immunofluorescence microscopy. (b) The microscopic appearance of minor salivary glands from non-SjD and SjD patients. Note the multiple scattered lymphocytic foci, periductal fibrosis, and atrophy characteristic of SjD. (c) A heatmap of clinical features for the scRNA-seq patients exhibiting the intrinsic clinical heterogeneity of subject phenotypes (e.g., SjD, non-SjD sicca). (d) PCA of patients using five of the clinical features. Importantly, the second principal component of the PCA analysis divides the patients on autoantibody positivity for anti-SSA autoantibodies (SSA+). (e) Leiden clustering followed by manual annotation based on gene expression and UMAP embeddings enables granular distinctions between cell types. Cells clustered with Leiden clustering in scanpy are labeled along with manual embeddings based on cell expression and boundaries in UMAP embeddings in boxes. In total, 31 unique cell types, including seven types of seromucous acinar cells, were identified. Two PRR4- SMAC populations were combined into one “transitioning” population. (f) Cells by the diagnosis of the patient, with and without SjD. (g) Cells clustered by anti-SSA autoantibody positivity from patients.

We collected research biopsies for single cell RNA sequencing from 11 Sjögren’s and 14 nonSjD patients (Supplemental Table 1). After quality control, 94,000 single cells were collected and sequenced (Figure 1). Six thousand spots of spatial RNA-sequencing were collected from 38 patients: 19 with a diagnosis of Sjögren’s (SjD), 14 with sicca but without a diagnosis of Sjögren’s (non-SjD) and 5 healthy volunteers (HV) (Supplemental Table 2). These patients included clinical presentations with variation in age, sex, severity of disease and positivity for autoantibodies (Figure 1b). A principal component analysis (PCA) of clinical data from patients for which single-cell data was generated clearly discriminated between anti-SSA autoantibody positive (SSA+) and anti-SSA autoantibody negative (SSA-) patients along the second principal component (Figure 1c). Using the single-cell data to define cell types, 31 unique clusters were identified by Leiden clustering followed by manual annotation based on defined cell populations (Figure 1e). These included six unique types of seromucous acinar cells, two sets of mucous cells, and five groups of T cells (Figure 1e, Figure S1a,b). Some cell types were enriched in disease (Figure 1f) or in patients that were SSA+ (e.g., CD8+ Exhausted T cells, CD4+ T cells, B cells, and Natural Killer (NK) cells; Figure 1g, S1a). These unique and complementary datasets were helpful to establish an unbiased portfolio of alterations in the SjD-affected salivary glands and a foundation for discovering and testing important cell types, cell states, and cell interactions in SjD.

### Sjögren’s is defined by loss of a seromucous acinar cell population

**Figure 2:**
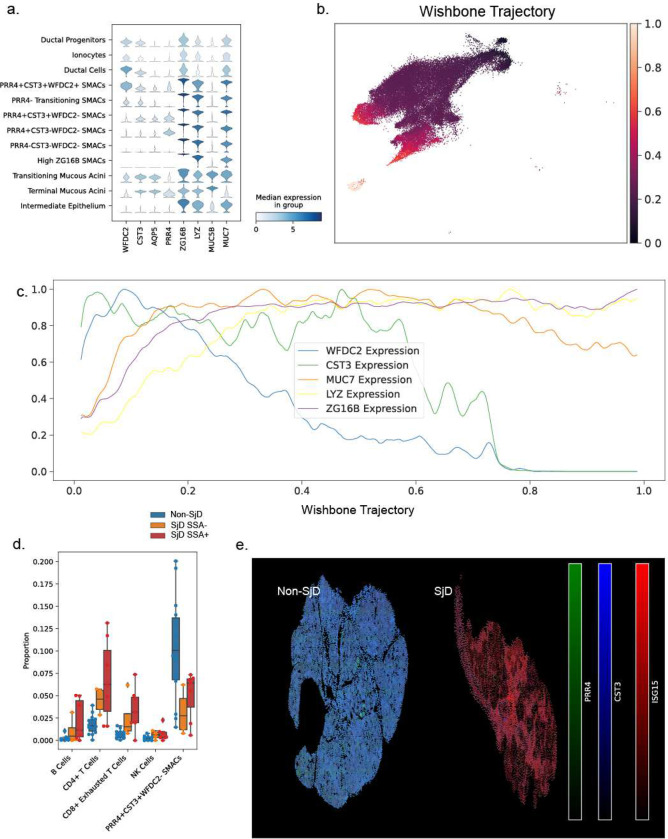
One seromucous acinar cell population represents the greatest loss of epithelium in disease. (a) Expression of acinar cell type markers in the scRNAseq data including in the six distinct populations of seromucous acinar cell types and two populations of mucous acinar cells. (b) Cell trajectory for ductal and seromucous acinar cells using wishbone, starting with the cell most positive for *KRT5*, a marker of progenitor state. The cell trajectory starts with a small population of ductal progenitors and proceeds along the ducts to the seromucous acinar cells, ending in the CST3− populations. (c) Gene expression (after log-transformation) for marker genes along the wishbone trajectory as a fraction of maximum expression. *WFDC2* expression is lost first, followed by *CST3*. *MUC7* expression peaks in the center and *LYZ* and *ZG16B* are highest in the most differentiated SMACs. (d) Statistically significant changes in cell proportions between SjD and nonSjD. PRR4+CST3+WFDC2− SMACs were the most abundant population in non-SjD patients and presented the greatest loss in SjD. (e) Representative images showing multiplex in-situ RNA hybridization of acinar and disease marker genes in salivary glands.

The principal target cells and antigens have yet to be elucidated in SjD. To study the SjD-specific effects on epithelial cells, we used our single cell analyses and spatial transcriptomics (Figure 1, Figure 2a) to study the composition and architectural changes in the context of SjD. Six distinct cell populations expressing key seromucous acinar cell (SMAC) markers were identified using Leiden clustering followed by manual annotation (Figure 2a). The most “stem-like” epithelial cells identified were a subpopulation of cells clustering within the ductal cell cluster subsequently named ‘Ductal Progenitors’ (*KRT5*)^32^. Starting from this cell, Wishbone was used to calculate a trajectory score across all ductal and seromucous epithelial cells (Figure 2b). The gradient in trajectory score from ducts to more-terminally-differentiated acinar cells is consonant with developmental tubulogenesis of the exocrine gland and the ability of duct cells to regenerate acinar tissue. Exploring the SMAC cells across this trajectory score, genes of ductal differentiation fall in expression (*WFDC2*) and genes associated with seromucous acinar function increase in expression (*ZG16B*, *PRR4*; Figure 2c). *MUC7*, a seromucous acinar protein, is highest in the *CST3+* SMACs. The cells seem to follow two trajectories terminating in *PRR4* high and *ZG16B* high cell clusters. When examining the differences in proportions of cells in the salivary glands of SjD patients, the *PRR4+CST3+* acinar cell cluster, which constitute the largest single population of SMACs in nonSjD glands, are significantly reduced in SjD (Figure 2d) while the other acinar cell populations remain statistically unaffected (Figure S1a).

scRNAseq reveals striking and underappreciated heterogeneity of the acinar cell composition of the salivary glands and that in SjD seems to result in preferential loss of SMACs (Figure 2a,d; Figure S1a,b)^33^. To confirm changes in heterogeneity from scRNAseq among the salivary glands cell populations, we used hybrid fluorescent 12-plex ISH/3-plex immunofluorescent spatial phenotyping. We applied markers that discriminate SMAC clusters (*MUC7*, *MUC5B*, *ZG16B*, *WFDC2*, *CST3*, and *PRR4*); and applied canonical markers of salivary differentiation (*BPIFB2, PIP, AMY1A*), markers of response to Type I IFN (*ISG15, B2M*), and protein markers (pan-cytokeratin [pCTK], CD45) (Figure 2a,d, Figure S3). Following recursive fluorescent whole slide imaging and registration, cells were segmented and phenotyped. Distribution of ISH quantifications separated cells from SjD and non-SjD patients (Figure S2). Finer-grained clustering separated patients on severity of disease presentation (Figure S3). Cells from patients with SjD were uniformly higher in quantification of Type I IFN response genes (i.e., *ISG15*, *B2M*) and lower in quantification of PRR4 and CST3 (Figure 2e) confirming our scRNAseq data.

### Surviving epithelium is undergoing mitosis in patients with SjD

**Figure 3:**
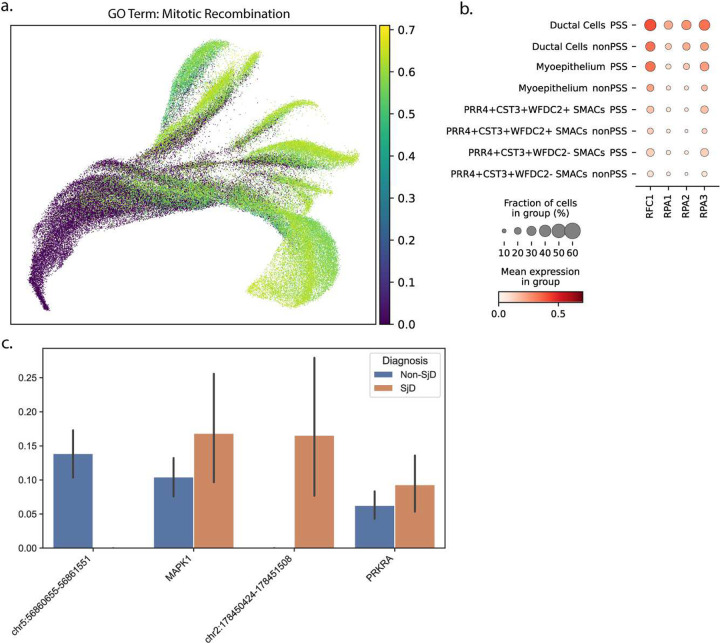
Clinical research investigations for 25 subjects included comprehensive oral, rheumatological, and ophthalmologic investigations applying American College of Rheumatology 2016 Sjögren’s Disease Classification Criteria including salivary gland biopsies on all subjects. Single-cell RNA-sequencing data from 94,227 cells was gathered across 25 patients with and without SjD disease. (a) A cartoon depicting the sample collection from subjects and the creation of scRNA-seq and spRNA-seq libraries. Histological interpretations were rendered on all subjects and patients’ glands. Additional subjects, not included in the scRNAseq analyses, were used for flow cytometry, 10X Visium spatial transcriptomics, multiplex fluorescent in situ hybridization (PhenoCycler-Fusion), and multiplex immunofluorescence microscopy. (b) The microscopic appearance of minor salivary glands from non-SjD and SjD patients. Note the multiple scattered lymphocytic foci, periductal fibrosis, and atrophy characteristic of SjD. (c) A heatmap of clinical features for the scRNA-seq patients exhibiting the intrinsic clinical heterogeneity of subject phenotypes (e.g., SjD, non-SjD sicca). (d) PCA of patients using five of the clinical features. Importantly, the second principal component of the PCA analysis divides the patients on autoantibody positivity for anti-SSA autoantibodies (SSA+). (e) Leiden clustering followed by manual annotation based on gene expression and UMAP embeddings enables granular distinctions between cell types. Cells clustered with Leiden clustering in scanpy are labeled along with manual embeddings based on cell expression and boundaries in UMAP embeddings in boxes. In total, 31 unique cell types, including seven types of seromucous acinar cells, were identified. Two PRR4- SMAC populations were combined into one “transitioning” population. (f) Cells by the diagnosis of the patient, with and without SjD. (g) Cells clustered by anti-SSA autoantibody positivity from patients.

To understand disease- and cell-type-specific pathway utilization, we hypothesized that scRNAseq data could be clustered based on indices of gene expression. Expression data was converted from log-normalized counts to Z-scores to make contributions between genes equivalent and summed across annotated indices to form S-scores. A UMAP built on these S-scores split cells by an index of recombination linked to mitosis (Figure 3a). Genes involved in mitosis are more highly expressed in epithelial populations in SjD, including in the seromucous acinar population lost in disease (Figure 3b). To investigate changes in chromatin accessibility upstream of gene expression, 10x Multiome data was generated on a limited set of available samples (n=6, Table 5). The most differentially open chromatin in PRR4+CST3+WFDC2− seromucous acinar cells was found at chr5:56860655–56861551 and linked to higher expression in MAPK1, a gene associated with cell division, and the most differentially closed chromatin was at chr2:178450424–178451508 and linked to PRKRA, a gene involved in the interferon response pathway (Figure 3c). Expression of both genes was higher in seromucous cells in SjD.

### Cells grouped by index expression suggest two paths to T cell dysfunction.

**Figure 4:**
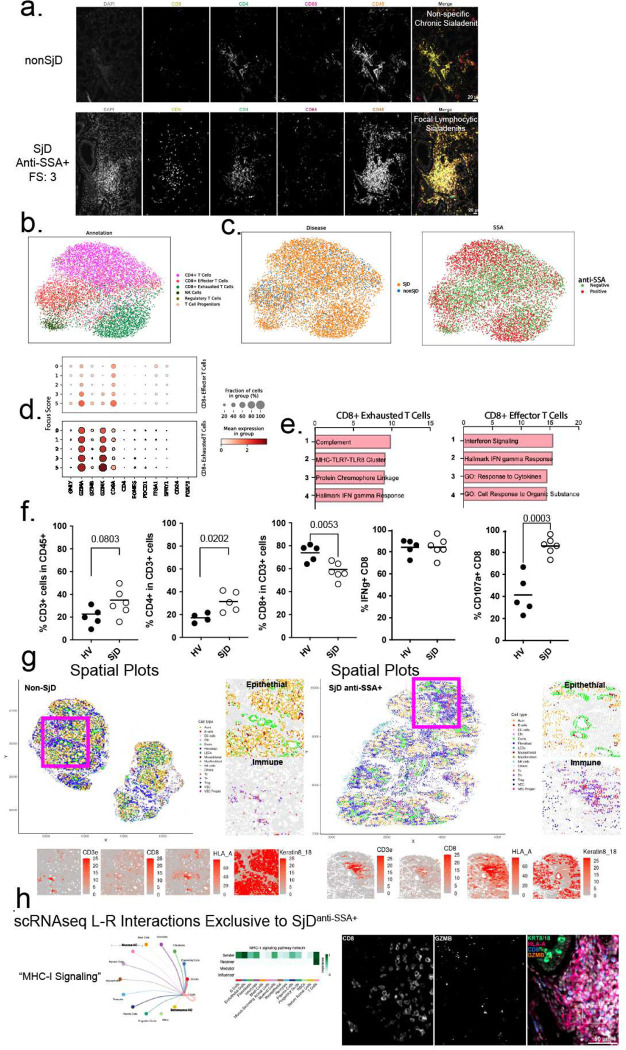
CD8 T Exhausted T cells are enriched in the immune infiltrates of SjD and exhibit an effector phenotype. (a) Highly multiplexed (4/35-plex shown) immunofluorescence microscopy shows distribution of CD4+ and CD8+ T cells, as well as, CD68+ macrophages in the glands of SjD and nonSjD. (b,c) scRNAseq UMAPs of expression using only T cells, with disease diagnosis and anti-SSA positivity visualized. T cells from anti-SSA positive individuals cluster on the periphery while SjD-positive T cells are distributed throughout. (d) Expression of key T cell genes in exhausted and effector CD8+ T cells across disease severity. (e) Single cell pathway enrichment analysis shows similar activated profiles between *GZMK*+ CD8+ Exhausted T cells and CD8+ Effector T cells in SjD. (f) The potential for degranulation and cytotoxicity of T cells was measured *ex vivo* using flow cytometry-based T-lymphocyte cytotoxicity assay. CD45+ immune cells were dissociated from patients’ salivary glands (nonSjD: n=5 patients; SjD: n=6 patients). (g) Spatial plots of segmented and phenotyped multiplex immunofluorescence data confirm alterations in the cellular arrangement of the glands, and highlight T cells at the epithelial interface in SjD. (h) CellChat Ligand-receptor analysis of scRNAseq data shows enriched signaling pathways (e.g., “MHC-I signaling”) is specific to anti-SSA+ SjD with connections to multiple cell types and the physical location of CD8+ T cells to immune-involved epithelia is shown.

The scRNAseq data was transformed to S-scores of gene indices as described in the previous section. Clusters represent a composite of previously named cell types (Figure SF4a-c). Given these clusters encompassed multiple cell types and were characterized by a regulatory program or shared function — exhibiting contractility, or undergoing cell division — they remained unnamed (Figure S4c). Like the clusters based on expression (Figure 1e-g, Figure S1a), clusters based on index scores were enriched for clinical features of interest in SjD (Figure S4c-f, Figure S5a). Cells from nonSjD and SSA− patients’ cells shared expression of indices, indicating their lack of dissimilarity —sensory taste perception and pathological glycosylation. Conversely, cells from SSA+ patients were associated with many enriched pathways involving effector immune cell function, antigen processing, and IFN signaling (Figure S5f,g). To illustrate this more clearly, a 21-gene Type I interferon-stimulated genes score was constructed and found to be higher in every annotated cell type in SSA+ patients, irrespective of SjD status (Figure S5c,d), and was found to be highest in endothelial cells, macrophages, dendritic cells, and ducts. Calculating a ratio of cells in each clinical feature, two index clusters have notable enrichments with disease or SSA+ (Figure S4e,f). These cells were almost exclusively identified previously as either T-lymphocytes or antigen-presenting cells (i.e., B-lymphocytes, dendritic cells, or macrophages; Figure 1e, Figure S4b-e, Figure 4a). Comparing the indices directly, indices associated with T cell activation scored higher in the SSA+ cluster; no indices were significantly enriched in the Sjögren’s-diagnosed cluster (Figure S5b).

### CD8-GZMK T cells are enriched in SjD and exhibit effector functionality.

The data above highlight important cell types in SjD pathogenesis. The vast majority of these cells are found within dense collections of inflammatory cells termed “foci”, and represent the characteristic inflammation of SjD, focal lymphocytic sialadenitis (Figure 1b, Figure 4a). SjD immune foci are characterized by predominantly lymphocytic infiltrates composed of T- and B-lymphocytes. To visualize and quantify the expression, measure the function of T cells infiltrating SjD salivary glands, and to understand how these cells interact and organize across the gland, we analyzed spRNAseq, scRNAseq, and flow cytometry data. T cell subclusters are annotated based on their expression profiles into CD4+ T cells (*CD40L*), Regulatory T cells (*FOXP3*), Progenitor T cells (*MKI67*, *CD25*), Exhausted CD8-T cells (Tex; *CD8A, GZMK, PDCD1*), Cytotoxic CD8-T cells (Tc; *CD8A, GZMB*), and NK cells (*NKG7, GNLY)* (Figure 4b). The most disease-enriched immune cell type in SjD salivary glands are Tex (4.75-fold enriched Tex, q = 0.0009; Figure 4b,c; Figure 1e; Figure S1a). Focusing on this population we wanted to understand their role in salivary gland pathology in SjD. Clustering by disease feature shows enrichment of T cell subtypes in SjD and anti-SSA+ specific clustering along the periphery of the individual subclusters (Figure 4c). Looking at gene expression in T cells, *GZMA* and *GZMK* mRNA were higher in both SSA− SjD and SSA+ SjD, while *GNLY* and *GZMB* exhibited lower in expression in the same patients, indicative of the shift towards more *GZMK*+ Tex in the salivary glands (Figure 4d). Tc and Tex displayed similar pathway activation, with greater activation in SSA+ patients (Figure 4d,e). Although *GZMB+* Tc cells are numerically increased in SjD, they are fewer than Tex and not significantly enriched; however, they do display similar pathway enrichment (as Tex).

To test the cytotoxicity of T cells infiltrating the salivary glands, we isolated immune cells from patients’ salivary glands (nonSjD: n=5 patients; SjD: n=6 patients) and performed an *ex vivo* T-lymphocyte cytotoxicity assay to measure intracellular cytokine expression and surface expression of CD107a. Expectedly, SjD salivary glands show marginally increased proportions of T cells in the glands (p=0.08; two-sided *t-*test), with increased CD4+ T cells (1.6-fold, p<0.05; two-sided *t*-test) and decreased CD8+ T cells (0.8-fold, *p*<0.01; two-sided *t*-test) ratios (Figure 4f). A high proportion of CD8+ T cells from salivary glands express IFN**γ**; however SjD glands show increased proportions of CD8+ T cells positive for surface CD107a in response to PMA/ionomycin stimulation, indicative of enhanced degranulation (e.g., cytotoxic potential; 2.1-fold, *p*<0.001; two-sided *t*-test; Figure 4f). While it was not possible to separate Tc and Tex subsets from human salivary glands for this assay, it supports that both Tc and Tex exhibit enhanced effector function in the glands of SjD patients.

### Spatial phenotyping demonstrates Sjögren’s-Specific Cellular Interactions.

Multiplex spatial phenotyping is an emerging technology ideally suited to map the positions and interactions of cells and to provide in the salivary glands of SjD and healthy patients. Representative salivary gland FFPE sections containing 2–5 glands from SjD (n=5) and nonSjD (n=5) patients were analyzed using 35-plex spatial phenotyping platform to identify 16 cell types: Th, Treg, Tc, NK, DC/mac, LEC, VEC, progenitor VEC, myofibroblasts, fibroblasts, myoepithelial, ducts, acini (Figure S6a, *Supplemental Methods*), although this initial panel was unable to discriminate all cell types resolved in scRNAseq (Figure 1e; e.g., clear separation of seromucous from mucous acinar cells). Spatial phenotyping corroborates the general scRNAseq-based cellular proportion changes including: reduced numbers of acini and myoepithelial cells, greater proportions of ducts and immune cells including statistically more Tc (2-fold; *t*-test, p<0.05) and Th (2-fold; two-sided t-test, *p*<0.05), and marginally more Treg (5-fold; two-sided t-test, p=0.052) (Figure S6b). Th and Tc cells in SjD express higher CD45, IFNγ, HLA-DR, HLA-A, CD107a, and reduced expression of GZMB and immune checkpoint markers, PD-1 and IDO1 (Figure S6c). SjD ducts, acini, and myoepithelial cells show greater HLA-A and HLA-DR expression. Importantly, SjD acini show altered expression of acinar markers: increased CD66 (greater mucous than seromucous expression), lower Galectin-3 (greater seromucous than mucous expression), and decreased Ki67 (Figure S6c). To directly visualize the arrangement of immune cells in the salivary glands of SjD, we constructed spatial plots of the identified cell types. Consistent with above, SjD glands exhibit reduced acinar density and dense immune cell clusters (i.e., foci), circumscribing ducts and inter-acinar spaces (Figure 4g). Th, Tc, B cells congregate around epithelial structures with increased expression of HLA-A at the immune interface (Figure 4g). Using neighborhood analysis, SjD increases the interactions between multiple immune cell types (e.g., B, DC, Treg, Tc; Figure 4g, Figure S6d). At the immune-epithelial interface, acini show enhanced interactions with Treg, Th, DC, and Tc; ducts exhibit increases in B, Treg, Th, DC, and Tc; and myoepithelial cells show increased interactions with DC, TC, Th, but loss of interactions with other cell types (e.g., acini, ducts, stromal, and endothelial cells) (Figure 4g, Figure S6d) illustrating the complexity of cell interaction changes in SjD.

Concentrating on T cell interactions in SjD, we performed receptor-ligand analysis of our scRNAseq data (Figure 1c) using CellChat. Examining interactions that were found in anti-SSA+ SjD but absent in anti-SSA− SjD show “MHC-I signaling” as the most dominant inferred interaction in SSA+ SjD salivary glands (Figure 4h). Additional SSA+ SjD-specific receptor-ligand interactions included “CD22” and “BAFF” (Figure S6e), as well as “GRN”, “GAS”, “SELL”, “KIT”, “ICAM”, and “ADGRE5”. These results support that autoantibody-positive disease exhibits signaling centered on T cell/B cell signaling pathways (costimulation and maturation of B cells [CD22, BAFF], the Tc immunological synapse [GRN], and gamma-associated sequences [GAS], as well as, pathways involved in signaling infiltration of lymphocytes from the blood to peripheral organs [SELL, ICAM] (Figure 4g, Figure S6e,f). These results highlight the importance of effector (Tc) interaction with target cells (ducts and acini) and further support the biological distinction between SSA+ from SSA− SjD in the glandular pathology. What remains to be answered is what is the potential effector function of these SjD-enriched GZMK-Tex cells in the salivary glands?

### GZMK Drives Type I IFN Signaling in Salivary Epithelial Cells.

Very little is known about how *GZMK*+ CD8-Tex cells could exert an effector function or contribute to SjD pathology. Effector engagement with target cells occurs at the immunological synapse whereby a regulated exocytosis of cytotoxic granules, containing perforin-1 and granzymes, induce target cell effects including apoptosis. Unlike Tc cells that secrete cytotoxic granules containing GZMB, other granzymes (e.g., GZMA, GZMK) have unique targets and downstream functions. Specifically, GZMK does not directly induce caspase-dependent apoptosis^27^. Alternately, cytosolic GZMK has been shown to cleave BID in the mitochondrial membrane to drive the generation of reactive oxygen species (ROS) and potentially, caspase-independent cell death^27^. We hypothesize that in addition to the potential generation of ROS, cytosolic GZMK may affect mitochondrial membrane permeability promoting the relocalization of mitochondrial nucleic acids to the cytosol and the initiation of the pathogen recognition response (PRR)^28,29^. In the context of autoimmunity, epithelial cells expressing autoantigenic peptides on their MHC-I (Figure 4, Figure S6) may initiate effector cell engagement of Tex cells to drive subacute effects on inflammatory signaling thereby explaining 1) minimally increased apoptosis in the glands (i.e., the immunopathology does not seem to be directly Tc), and 2) the canonical increased Type I IFN signature in SjD-affected ducts and acini. This raised important questions including regarding the arrangement of *GZMK*+ CD8-Tex cells in the gland and their role in SjD pathogenesis.

**Figure 5:**
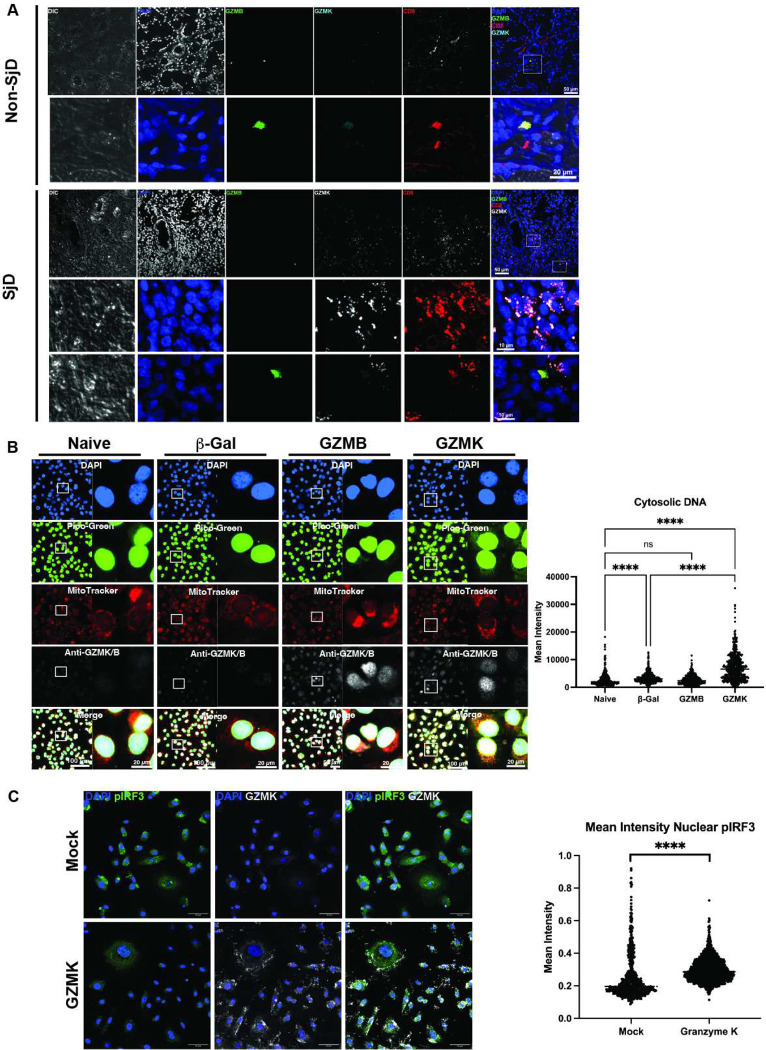
GZMK drives cellular innate immune signaling in SjD. (a) Multiplex immunofluorescence of salivary glands sections from SjD and nonSjD hybridized with anti-GZMK, -GZMB, and -CD8. Immune infiltrates enriched with GZMK+CD8+ T cells in close proximity to acinar structures and ducts are shown. (b) Fluorescent confocal microscopy was used to measure cytosolic mitochondrial DNA after GZMK protein transfection. The transfection of recombinant GZMK and GZMB were monitored by their His tag. Image analysis demonstrates increased mtDNA in the cytoplasm. (c) Multiplex immunofluorescence microscopy shows cytosolic transfection of GZMK drives phosphorylation IRF3 (pIRF3) and nuclear translocation in pSGEC.

We next confirmed the location and proportion of GZMK+ and GZMB+ CD8-T cells in the peri-epithelial immune infiltrates using immunofluorescence microscopy. GZMB+ CD8-T cells were fewer in number in the infiltrates and more distant from epithelial structures in SjD (Figure 4h, Figure 5a); GZMK+ CD8-T cells were more numerous and reside in close proximity to, or within, involved ducts and acini (Figure 5a). To model the effector activity of cytotoxic granules containing GZMK from Tex on targeted epithelia in SjD, we transfected GZMB and GZMK into the cytosol of human acinar cells (AC cells; Figure S7a). Transfection of GZMK did not induce apoptosis by staining with Annexin V/PI flow cytometry (Figure S7a); transfection of GZMB increased the amount of dead/floating cells in the supernatant, but not significant differences by flow cytometry. Western immunoblot analysis of GZMK shows the dose-dependent differences in GZMK protein levels in HSG cells (Figure S7b), and shows the time-dependent presence of cytosolic GMZK and GZMB protein in NS-SV-TT-AC cells out to 48 hours (Figure S7c). Using immunofluorescence microscopy, GZMK transfection, but not GZMB, results in mtDNA relocalization (loss of collocation with MitoTracker) to the cytosol (2.6-fold, *p*<0.0001; ANOVA; Figure 5b) in acinar cells. Correlation analysis of protein within the cell, labeled by His tag, shows that GZMK, but not GZMB, correlates with the presence of measurable cytosolic DNA. Cytosolic GZMK results in an increase in pIRF3/IRF3 ratio suggesting IRF Pathway activation and Type I IFN signaling (Figure S7d). The ability for GZMK to drive cytosolic mtDNA in reporter cells was confirmed using fluorescent microscopy of THP1-macrophages (Figure S7e). To show that GZMK-induced cytosolic mtDNA drives Type I IFN through PRR^28,29^, we used reporter cell lines to show IRF Pathway activation and Type I IFN responses, (1.6-fold, p<0.0001; ANOVA; Figure S7e) and Type I IFN response (1.3-fold, p=0.023; ANOVA) (Figure S7f), respectively. To further confirm this effect, we transfected GZMK into the cytosol of human primary salivary gland epithelial cells (pSGEC); GZMK induced cytosolic mtDNA and nuclear translocation of pIRF3 (1.2-fold, p <0.0001; two-sided *t*-test; Figure S7g) in pSGECs.

However, in furtherance of these results, we wanted to understand if Tex GZMK can be taken up by target salivary epithelial cells. To answer this question, we measured the presence of cytosolic effector granzymes in target epithelial cells. Using healthy and SjD patients, we used patient-matched primary salivary gland epithelial cells co-cultured with expanded CD8 T-lymphocytes at various target:effector ratios (1:2.5–1:20). We show that GZMK secreted from CD8 T-lymphocytes is taken up dose-dependently by target epithelial cells. Based on this and our data above, this suggests that Tex, like Tc, can exert effector function in the SjD salivary gland, yet alternatlely do not seem to directly induce cytotoxicity but instead activate cellular innate immune signaling through PRR/nucleic acid sensing pathways. *GZMK+*Tex-like cells have been reported to be involved in multiple target organs (e.g., kidneys, synovium) in autoimmune diseases^27,30^. Contemporaneous to our analyses, *GZMK+*Tex-like cells were found to be gland-enriched and clonally-expanded in SjD patients’ glands, possibly suggesting a salivary-specific autoantigen^31^. Adding upon this foundation, our results explain a unique effector function of these cells by demonstrating the potential for autoreactive *GZMK+*Tex cells to secrete GZMK on target cells to drive Type I IFN immune signaling in the salivary gland epithelia. This may partially explain Type I IFN signaling characteristic of SjD including in duct and acinar cells and implicate these cells in modulating immune signaling in foci with sustained antigen engagement. In our data, this effect is enhanced in the SSA+ SjD subjects. However, the real-time direct observation of these events in situ has not yet been possible.

### Spatial transcriptomics identifies cell interactions and architectural alterations in SjD.

**Figure 6:**
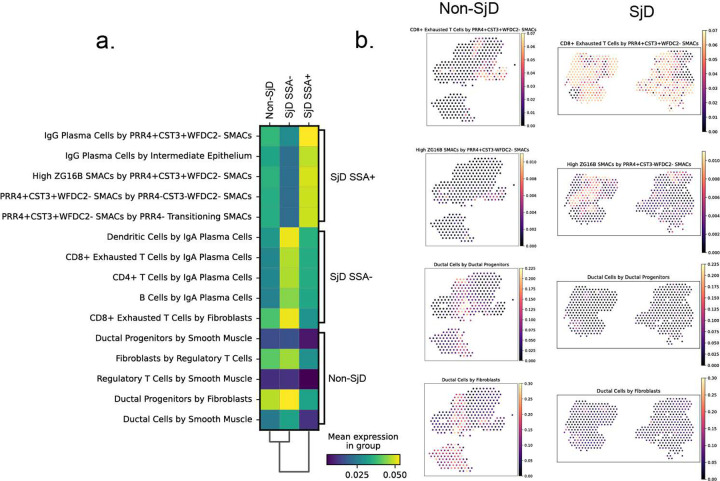
The quantification of cell types per spot according to Cell2Location. (a) Cell cooccurrence scores most different in disease. Immune cell colocalization with the *PRR4+CST3+WFDC2*− seromucous population is highest in SSA+ Sjögren’s patients. (b) Cooccurrence score spatial plots depict the altered cooccurrences between cell types including loss of cooccurrence of ductal cells with fibroblasts and increased cooccurrence of ductal cells with ductal progenitors, CD8+ Exhausted T cells with *PRR4+CST3+WFDC2*− SMACS, and high ZG16B SMACS with *PRR4+CST3-WFDC2*− SMACS.

Important disease and tissue-specific changes in cellular composition and gene expression state changes can be gleaned from single cell approaches in autoimmune diseases. Although high resolution at the single cell level, this comes at the expense of losing important cell interactions in the tissue context which can only be inferred bioinformatically. To determine cell interactions, spatially-encoded RNA-sequencing (spRNAseq) was performed (Figure S8a). Within-spot cell proportions were estimated using Cell2Location and expression patterns from scRNAseq (Figure S8b). Structural characteristics of the gland, at a resolution of 55 μm, were compared between disease and health and recapitulate typical architecture of the glands. The differences in within-spot cell proportions reveal important disease-specific changes in cellular interactions such as the absence of Tregs with collocated ductal progenitor cells, increased association between immune cell types within lymphocytic foci, periductal fibrosis, and heightened contact between seromucous acinar cell types (Figure S8c,d). Scoring cooccurrence for all cell types, and comparing SjD to non-SjD supported these differences, highlighting a notable increase in within-spot cooccurrence of CD8+ exhausted T cells and *PRR4+CST3+WFDC2−* seromucous acinar cells (Figure 6). Colocalizations also tracked changes in architecture of the gland, such as the loss of contacts between fibroblasts and ducts and the increased contacts between different types of acini.

## DISCUSSION

Building on our prior work,^34,35^ in this study we have applied data gained from single cell RNA-seq and spatial RNA-sequencing to identify a unique pathogenic population of *GZMK*+CD8 Tex cells that appear to be central to the development of Sjögren’s Disease.. This conclusion was reached by using the single-cell data to identify unique clusters, including seven unique seromucous acinar cell types not previously annotated and five different T cell types. These clusters were used to quantify the percent of cell type in each spot in the spatial data, providing evidence of cell-cell colocalization changes in disease and health. Spatial phenotyping and *ex vivo* cellular assays further confirm that SjD-enriched *GZMK*+CD8 Tex cells are enriched in the glands, colocate with ducts and acini, and exhibit cytotoxic potential. SSA+ and Sjögren’s diagnosis shared a group of associated indices, including NK- and T-cell-mediated cytotoxicity and antigen presentation, and a list of indices lower in expression. However, the manifold built on index S-scores revealed two distinct paths to T cell dysfunction in Sjögren’s Disease, one associated with autoantibody positivity, and one negative. Changes characteristic of SSA+ included increased T cell activation, antigen presentation accompanied by changes in MHC expression and an upregulation across the gland of genes inducible by interferon signaling; these changes were absent in SSA− disease confirming the distinct biological heterogeneity between antibody positive and antibody negative disease.

For the first time we show both direct and indirect evidence that *GZMK+*CD8 Tex cells may exert potent and a specific immune response in target cells that express markers of acinar differentiation but lie transcriptionally nearest to a progenitor duct-like cell type origin. This effect ultimately reduces the proportions of terminally differentiated acinar cells, with appreciably less effect upon the mucous acinar cells of the glands. This can be appreciated microscopically whereby mucous acinar cells and ducts dominate the typical histopathological profile in Sjögren’s disease MSG biopsies.

What was definitional of disease in both SSA+ and SSA− Sjögren’s patients was a loss of *PRR4+CST3+WFDC2*− seromucous acinar cells characterized by the highest levels of expression of *MUC7*, which fell from the largest single cell population in nonSjD patients to representing less than half that level in SjD. The proportions of other SMAC populations remained statistically unchanged by disease; yet exhibited lower proportions. This can be explained by gland atrophy which is a feature of more longstanding disease. Moreover, it is not possible to quantify the cells that were lost prior to biopsy. One hypothesis for this vulnerability comes from animal models of autoimmune thyroid disease where CD8 T cells remove hypersecreting mutants from glandular epithelia in a theory called “autoimmune hypersecreting mutants (AHSM)”^36^. In Sjögren’s Disease, we surmise that autoreactive *GZMK*+CD8+ T cells may selectively remove or restrain the differentiation of transitioning seromucous acinar cells with high levels of secretion. Unsurprisingly for this autoimmune disease, invasive lymphocytes replaced *PRR4+CST3+WFDC2*− seromucous acinar cells nearly one-to-one. Surviving members of this seromucous acinar cluster in Sjögren’s Disease individuals have a distinct gene expression pattern that offers clues to the vulnerability of this population, with cells from non-Sjögren’s individuals having higher levels of expression of indices associated with development and proliferation which may have led to sensitivity in the Sjögren’s immune microenvironment. Looking at cell-cell type correlations within spots in the spRNAseq, negative correlations between unrelated cell types in the non-Sjögren’s individuals gave way to zero or low positive correlations in Sjögren’s Disease, suggesting a higher prevalence of interactions between previously distinct cell types. Differential colocalization scores revealed a pathogenic adaptive immune infiltrate adjacent to vulnerable seromucous acinar cells suggesting effector cell targeting of putatively antigen-expressing epithelial cells.

In conclusion, Sjögren’s Disease is a chronic systemic autoimmune disease characterized by loss of exocrine gland function. Using multiple new transcriptomic and proteomic datasets, we identify both the most sensitive target cells in the salivary epithelia of Sjögren’s Disease patients and an important population of *GZMK*+CD8+ T cells defined by a novel effector function of this cell type: altering mitochondrial membrane permeability and eliciting cellular innate immune signaling. These results provide a potential rationale for non-viral Type I IFN signaling from the affected epithelia in Sjögren’s Disease.

## LIMITATIONS:

By taking a sample at a single time point this study represents a snapshot of dysfunctional glands and does not explore how Sjögren’s disease progresses. The disease duration in each patient can only be roughly estimated based on symptom prevalence. Some of our data suggest that pre-clinical disease (those with SSA+ without satisfying classification criteria) exhibit altered pathway utilization similar to disease-affected patients and may provide clues about “pre-clinical” disease. Moreover, undiagnosed comorbidities, changes in the microbiome, changes in hormone signaling, changes in metabolism and so on represent blind spots of variables that were not measured and cannot be easily controlled. Gene expression changes result from changes in histone modification, DNA accessibility, and regulatory element methylation, none of which were measured as part of this study. Protein quantification of a limited set of gene products was performed, mostly for immune cells and state markers. Despite sampling more than 90,000 unique cells with scRNAseq across our patients, certain cell types known to be present in the salivary gland, most notably nervous tissue, are not recovered by this method of single-cell library preparation. To overcome limitations associated with modest numbers of cases, multiple orthologous (e.g., spatial proteomics, spatial transcriptomics) and experimental approaches (in vitro assays using multiple cell lines including patient-derived primary cells) were employed. PhenoCycler-Fusion spatial phenotyping, in this dataset, exhibited limited ability to segment in the densest immune infiltrates, attenuating the true number of inflammatory cells present in the glands and the interactions amongst those cells. Thus, direct comparisons between spatial phenotyping and scRNAseq-based shifts in cellular proportions are not possible. However, the directions of the shifts are maintained, thus spatial phenotyping differences may be of lower magnitude albeit with maintained tissue context representing a potential trade-off between resolution and in situ location. While not a limitation, per se, our GZMK transfection experiments, using multiple cell lines (AC, THP-1 macrophages, and HSG, we were not able to reliably induce detectable ROS or cell death (early or late apoptosis) up to the maximum of what we could transfect into cells. This may reflect the cell lines tested or that the specific effects on targeted cells may be different (e.g., fibroblasts or salivary epithelial cells). This highlights opportunities to further explore the role of granzymes (e.g., Granzyme B, Granzyme A, Granzyme K) on intercellular innate immune signaling in a cell-type specific contexts.

### Online Methods:

#### Clinical Evaluation of Patients.

Research participants were consented to NIH-approved IRB protocols (15-D-0051, NCT00001390; PI-Warner) prior to any study procedure in accordance with the Declaration of Helsinki principles. All participants were evaluated and classified according to 2016 American College of Rheumatology (ACR) and the European League Against Rheumatism (EULAR) classification criteria^9^. Comparator tissues included subjects (nonSjD) who were otherwise healthy, but did not meet 2016 ACR-EULAR criteria. All subjects were screened for evidence of systemic autoimmunity and received comprehensive oral, salivary, rheumatological, and ophthalmological investigations^37^. All subjects received salivary gland biopsies and tissues were organized and submitted for histopathological assessment by a board-certified anatomical pathologists (DEK). Additional tissues for research were placed in ice-cold RPMI for subsequent single cell, or oriented and embedded in OCT and flash-frozen in a slurry of 100% ethanol in dry ice. Frozen tissues were stored at −80 °C.

#### Minor salivary gland tissue dissociation

Minor salivary glands were collected from participants who provided informed consent on NIH protocols 15-D-0051 (PI: Warner). Tissues were biopsied following standard methods and immediately placed in ice-cold RPMI. Tissues were sharply dissected into 1–2-mm pieces and then dissociated using the Miltenyi Multi-tissue Dissociation Kit A using the Multi_A01 in C-type tubes at 37 °C in an OctoMACS tissue disruptor using heated sleeves. Crude single-cell suspensions were serially filtered through 70- and 30-μm filters and rinsed with 1× Hanks’ buffered salt solution. Cells were centrifuged at 300g for 10 min at 4 °C and washed with 1× Hanks’ buffered salt solution. Cell counting and viability were determined using a Trypan blue exclusion assay. Suspensions with greater than 75% viability were used for subsequent sequencing.

#### Cell type analysis and further annotation.

Single-cell suspensions targeting approximately 5,000 cells were loaded onto a 10x Genomics Chromium Next GEM Chip B for capture. After cell capture, single-cell library preparation was performed using the 10x Chromium Next GEM Single Cell 3′ kit v3 (10x Genomics). The libraries were pooled and sequenced on four lanes of a NextSeq500 sequencer (Illumina), adopting the read configuration indicated by the manufacturer. 10x Genomics workflow was used for read processing. Cell Ranger v3.0.1 Single-Cell Software Suite was used for demultiplexing, barcode assignment and UMI quantification (http://software.10xgenomics.com/single-cell/overview/welcome). reads were aligned to the hg38 reference genome (Genome Reference Consortium Human Build 38) using a pre-built annotation package obtained from the 10x Genomics website (https://www.10xgenomics.com/). Samples were demultiplexed using the ‘cell ranger mkfastq’ function, and gene count matrices were generated using the ‘cellranger count’ function. Several previously published single cell captures from our laboratory are used in this analysis, but not comprehensively analyzed in aggregate, are included in this study (phs002446.v1.p1).

Analysis of the single-cell RNA-sequencing was done in Python 3.9.4 using numpy 1.21.2^38^, scipy 1.6.3^39^, statsmodels 0.12.2^40^, scikit-learn 0.24.2^41^, pandas 1.2.4^42^, anndata 0.8.0 and scanpy 1.7.2^43^. Figures were made using matplotlib 3.4.2^44^ and seaborn 0.11.1^45^. Cells were gated on 15% mitochondrial reads, 50% ribosomal reads, 5% hemoglobin gene reads and a minimum of 10 genes and 100 counts. A total of 94227 cells were retained for analysis. Nearest neighbors were identified using the BBKNN algorithm^46^ followed by UMAP manifold representation^47^ and Leiden clustering^48^. Leiden clusters were named by gene expression using known markers as well as scanpy’s rank_genes_groups tool, which in default mode implements a two-sided t-test with Benjamini-Hochberg false discovery rate correction on the log-normalized count matrix. In multiple cases a single Leiden cluster corresponded to multiple known cell types - when that occurred, clusters were divided based on gene expression and UMAP embeddings to generate new annotation classes. In one case, two seromucous acinar cell populations with nearly-identical patterns of expression were combined into a single “PRR4- Transitioning SMACs” population. When multiple clusters corresponded to a single previously-known cell type annotation, individual pairwise calls to rank_genes_groups were used to identify genes discriminating between these clusters. Differentially expressed genes were identified using a rank_genes_groups call between two clinical features.

#### Proportion changes.

To test changes in cell type proportions between clinical features the crosstab function from pandas was used to get a per-patient proportion for each cell annotation. T-tests between clinical features produced q-values after Benjamini-Hochberg multiple test correction, and q-values below 0.05 were deemed statistically significant.

#### Calculating Index S-scores.

A comprehensive set of gene indices including KEGG terms^49,50^, GO terms^51,52^, Reactome Pathways^53^, mSigDB Hallmark Pathways^54–56^, four pathways that represent senescence-associated secretory phenotypes^57^, a previously-published pathway of ECM factors involved in Sjögren’s fibrosis^35^ and a set of genes regulated by interferon was used to transform from a gene expression space to an index S-score space. Expression data from each gene were converted to Z-scores and this Z-scores matrix was multiplied by an encoding matrix representing the relationships between genes and indices. The new matrix has a S-score for each index which is a sum of the Z-scores of the genes that make up that index. These S-scores were then treated as if new gene expression data and a new UMAP was generated and Leiden clustering was performed. The relationship between the new and old clustering methods was quantified using the pandas crosstab function.

#### Trajectory analysis and differentiation.

Trajectory analysis in the single-cell was performed using the Wishbone algorithm on the subset of epithelial tissues. The initial cell was set as the cell with the highest MKI67 expression, a marker of stemness, which happened to be in the Ductal Progenitor population. All of the epithelial cells were arranged by Wishbone distance from the initial cell and the expression of key markers of epithelial identity were plotted after normalization with a 1000-cell cosine rolling window.

#### Spatial RNA-sequencing analysis.

The spatial RNA-sequencing data was analyzed in Python 3.9.7 using numpy 1.21.5, scipy 1.8.0, statsmodels 0.13.2, pandas 1.4.1, networkx 2.7.1^58^ and scanpy 1.8.2 and figures were made using matplotlib 3.5.1 and seaborn 0.11.2. Spots were gated identically to cells in the single-cell data and 11544 spots were used in the final analysis. A cell2location model^59^ was trained using the previous scRNAseq data and applied to the spRNAseq data followed by normalization to proportions. Correlations reported are Pearson’s ρ. The geojson package (2.5.0) was used to import manual annotations. Within- and next-spot correlations were calculated as well as a within-spot co-occurrence score C, which was quantified as:

$$C\left(\kappa_{1},\kappa_{2}\right)=\frac{\left(\kappa_{1}\;+\;\kappa_{2}\right)e^{-3\left|\kappa_{1}-\kappa_{2}\right|}}{2}$$

where $\kappa_{1}$ is the proportion of the first cell type and $\kappa_{2}$ the proportion of the second cell type within a spot. This score increases as the proportions increase and is higher if the proportions are close to the $\kappa_{1}=\kappa_{2}$ axis.

#### In vitro immune cell stimulation and flow cytometry.

Detailed methods for dissociating minor salivary glands and subsequent single cell RNAsequencing were published in Huang & Perez, et al., 2021^34^. Minor salivary glands were minced using a GentleMACS Tissue Dissociator (Miltenyi Biotec) and were enzymatically digested in a shaker incubator at 37°C for 40 min in RPMI medium containing 1 mg/ml Collagenase D, 1 mg/ml hyaluronidase and 50 U/ml DNase I (Sigma Aldrich). Cell suspensions were then passed through a 100 μm cell strainer and enriched for lymphocytes using a 40% Percoll density gradient centrifugation (GE Life Sciences). Cells were stimulated with phorbol myristate acetate and ionomycin (ThermoFisher) at 37°C for 4 hours in the presence of anti-CD107a (BioLegend), brefeldin A, and monensin (ThermoFisher) in Complete RPMI media containing 10% fetal calf serum (FCS), 1% Sodium pyruvate, 25 mM Hepes, and 2 mM l-glutamate (ThermoFisher). Cells were stained with various combinations of fluorochrome-labeled antibodies and Fixable Viability Dye eFluor 780 (purchased from BioLegend or ThermoFisher) in PBS containing 1% FCS and 1X Brilliant Stain Buffer (BD Biosciences) for 20 min at 4°C (Supplemental Methods Table 1). After fixation and permeabilization using Foxp3/Transcription Factor Staining Kit (ThermoFisher), intracellular cytokine staining was performed in PBS containing 1% FCS, 1X Brilliant Stain Buffer, and 1X Permeabilization buffer for 1 hour at 4°C. Samples were acquired using a FACSymphony cytometer (BD Biosciences) and analyzed using FlowJo software (FlowJo, LLC).

#### Multiplex proteomics (Phenocycler Fusion).

The multiplex was performed using 5 μm FFPE sections mounted on SuperFrost plus (ThermoFisher MA, USA), which underwent deparaffinization and rehydration. Slides were soaked in a Coplin jar with 1:20 AR9 buffer (Akoya Biosciences, MA – USA). The jar was seated in a pressure cook for 15 minutes at low pressure. Samples went to tabletop cooling for 30 min, followed by 30 seconds in deionized water and 100% EtOH for 3min. All slides went through pre-staining procedures by immersing the slides in the Hydration Buffer for 2 minutes and staining buffer for 20 minutes (Akoya Biosciences, MA – USA). The antibody cocktail for primary incubation was prepared using 4 blockers (G, S, J, and N), including 9.5 microliters of each in 362 μL of staining buffers. For each slide, we aliquoted 150ml and included 1 microliter of each of the antibodies (list below). The slides were removed from the staining buffer and accommodated in a humidity chamber (StainStray, Sigma-Aldrich, MO – USA), and primary incubation was done overnight on 4°C refrigerator. After incubation, the slides were followed by a post-staining fixing solution for 10 minutes. After fixation, sequential 1-minute PBS washes were performed, followed by an immersion in ice-cold MeOH for 5 minutes. The sections were treated with a 200 μL of final fixative solution for 20 minutes. Additional washes were performed to remove the final fixative solution. Slides were dried and mounted with the Akoya flow using a press that seals the flow cell/coverslip on the slides for 30 seconds.

The slides were removed from the press and soaked in a 1X PCF buffer (Akoya Biosciences, MA – USA). To prepare the PCF reporter wells, a 15 mL Falcon tube was initially covered with aluminum foil. Subsequently, into this Falcon tube, we introduced 6.1 mL of nuclease-free water, along with 675 μL of 10X PCF buffer, 450 μL of PCF assay reagent, and 4.5 μL of a concentrated DAPI solution that we had prepared in-house. This DAPI addition resulted in a final DAPI concentration of 1:1000. The reporter stock solution was then pipetted into 18 amber vials, for each slide, with each vial containing 235 μL of the solution. To each vial, we added 5 μL of reporter per cycle. The total volume per vial was either 245 μL for a cycle involving 2 reporters or 250 μL for a cycle with 3 reporters (see Supplemental Methods Table 2.).

A specific criterion was established for each cycle, allowing for a maximum of 3 reporters. These reporters were chosen from a pool of Atto550, AlexaFluor 647, and AlexaFluor 750, as deemed appropriate based on the experimental requirements (specific reporter FluorChannels and antibody barcodes in the list below). Distinct pipette tips were employed to transfer the contents of each amber vial into a 96-well plate. Vials containing DAPI were pipetted into wells within the H-row, while vials containing reporters were distributed into wells in other rows of the 96-well plate.

After completing the well-filling process, the wells were securely sealed using adhesive aluminum foil (Akoya Biosciences, MA – USA). Subsequent imaging procedures were conducted using a PhenoImager Fusion system, which was connected to a PhenoCycler (specifically, the PhenoCycler Fusion system from Akoya BioSciences). The imaging was performed with a 20X objective lens from Olympus.

The necessary solutions for instrument operation included ACS-grade DMSO from Fisher Chemical, nuclease-free water, and 1X PCF buffer with the addition of a buffer additive. This latter solution was prepared by mixing 100 mL of 10X PCF buffer and 100 mL of buffer additive with 800 mL of nuclease-free water.

#### Image acquisition and Segmentation.

A multi-step procedure was employed to analyze the protein multiplex images following image acquisition using Qupath^60^. The whole slide images were analyzed using the 16-bit image directly provided as the raw file from PhenoImager. Individual slides were evaluated by a trained pathologist (BM) for each of the 40 antibody markers to set manual thresholds and use as a comparison point for the computational assignment described in the next session. The images underwent segmentation using a pre-trained model based on Cellpose 2.0^61^. Segmentation was refined iteratively, utilizing 80 fluorescent images sourced from post-mortem biopsies. The model underwent multiple training iterations until precise delineation of cell expansion was achieved in both the acinar cells and ducts. Each of the minor salivary gland biopsies were segmented separately. Following nuclei-based cell segmentation, raw data was exported, and the number of pixel value units per cell and channel was quantified based on the threshold.

#### Multiplex Proteomics Image Analysis: Preparing cell type signature matrix.

The matrix has cell type markers in rows and cell types of interest in columns. An element of this matrix has a value 1 for every cell type marker considered to be highly expressed in the given cell type (i.e., column) and 0 otherwise. Markers distinguishing deeper levels of cell granularity (e.g., CD4 for Helper T cells which are a subclass of T cells, or CD8 for Cytotoxic T cells which are another subclass of T cells) assume values corresponding to their cell granularity level in the signature matrix (e.g., 2 for hierarchy level 2).

#### Cell type annotation using TACIT.

The TACIT algorithm takes two main inputs: a matrix consisting of z-normalized marker intensities for each cell, and the cell type signature matrix described above. To start, the two matrices are multiplied to derive the Cell Type Relevance scores (CTR) for all cell types in all cells. The CTR score becomes a linear combination of the intensities of markers defining the specific cell type in the cell. The higher the CTR score, the stronger the evidence that the cell is representing a given cell type. As next, TACIT examines the distribution of the CTR scores across all cells and conducts primary cell type assignments in multiple steps: (1) Using the z-normalized marker intensities, cells are first grouped into a large number of seed clusters, each of which averages 0.5% of total cells and represents a small but tight community of cells with highly similar overall marker profiles; (2) For each cell type, the distribution of the cluster median CTR scores across all seed clusters is plotted in ascending order; (3) Piecewise regression is then employed to identify breakpoints that separate data (here, clusters) into different linear trends over different data regions (here, cluster ranks). The piecewise regression model is fit to the data allowing for up to three breakpoints, and the model that best represents the data is chosen based on the Akaike Information Criterion (AIC) score; (4) Once breakpoints are determined, clusters are categorized into the “low relevance group (LRG)” and “high relevance group (HRG)”, with the former encompassing all clusters ranking below the lowest breakpoint, and the latter group encompassing clusters ranking above the highest breakpoint; (5) CTR value that minimizes classification error between LRG and HRG is determined and used as a cutoff to assign primary cell type. Steps (1) to (5) are performed on each considered cell type individually. A binary cell type matrix (CTM) is formed, where cells (in rows) are assigned 1 for all plausible cell types (in columns) whose CTR scores pass the cutoffs determined in step (5).

#### Mixed cell type deconvolution.

CTM columns representing lower granularity cell types (columns for T-helper cells or cytotoxic T cells) are updated in accordance with the individual marker positivity (CD4 or CD8, respectively, using the cited example) and an AND logical gate. The individual marker positivity (0 if below or 1 is above the cutoff) is derived as it was the case of CTR score described in step 5 above, except that this time CTR score is a single marker (not a linear combination of multiple markers). The updated binary CTM matrix will contain a fraction of cells uniquely defined (rows for which only once a value of 1 has been registered across the columns), with the remaining cells exhibiting multiple cell type assignments (multiple columns have a value of 1 in a row, indicating the assignment of various cell types to the cell in that row). To resolve the conflict, clean cells from each cell type involved in the mixture group together with the ambiguous cells will be projected onto the feature space composed of relevant markers only (i.e., those that define considered cell types). A cell with mixed identities is reassigned to the cell type exhibited by the majority of its k nearest clean cell neighbors using KNN classification approach. The cells with mixed identities are then reassigned to the most similar cell type of their closest neighbors using KNN classification approach. Uniform Manifold Approximation Projection (UMAP) is used for visualization and verification of the cell type assignment results.

#### Cell-cell interactions and neighborhood analysis.

PhenoCycler data from each individual tissue was processed with Squidpy (version 1.3.1)^62^ that describes cellular interactions as graphs with nodes representing individual cells and edges potential cellular interactions as determined by Delaunay triangulation. A 99th percentile distance threshold for each tissue was set to remove edges representing improbably long cell-to-cell distances. Non-deconvoluted cells (classified under “Unknown” cell types) were excluded from the analysis before performing Delaunay triangulation. An interaction matrix was constructed, with an element ai,j representing the number of edges shared between the cell type i and cell type j. Healthy interaction matrix is an aggregate of connections in healthy tissues, while disease interaction matrix is derived from all diseased tissues. The matrices were column-normalized and used for comparative analysis of cellular interactions between healthy and diseased tissues. The elements of the diseased interaction matrix was divided by the elements of the healthy interaction matrix, and the resultant fold-change interaction matrix was examined for differences in cell type interactions between the two conditions, with FC values above 1 indicating more pronounced interactions in diseased tissues, and FC values below 1 suggesting more interactions in healthy tissues. To visually represent these differences, a hierarchically clustered heatmap with Euclidean distance was used.

#### Cell Lines and Cell Culture.

THP1-Lucia ISG and IFN-α/β Reporter HEK 293 Cells were purchased (Invivogen, San Diego, CA, USA); the immortalized NS-SV-TTAC Acinar Cell line (aka: AC cells) were provided by Dr. Jay Chiorini. THP1 and 293 cells were cultured as previously reported in RPMI (Corning Cellgro, Corning, NY, USA, #15–040-CV) and DMEM (Gibco, New York, USA, #10313–021), respectively, supplemented with 10% FBS (Gibco, #A47668–01) and penicillin/streptomycin 100 U/mL, 2 mM L-glutamine, 100 ug/mL Normocin. The NS-SV-TTAC line was cultured in Defined K-SFM (ThermoFisher, #10744019), supplemented with Defined Keratinocyte-SFM Growth Supplement. The Human Salivary Gland (aka: HSG) was cultured in DMEM (Gibco, New York, USA, #10313–021) and EMEM (Quality Biological, Gaithersburg, MD, #112–018-101) at a (1:1 ratio), supplemented with 10% FBS and penicillin/streptomycin 100 U/mL, 2 mM L-glutamine. The immortalized HSG cell line is known to be HeLa contaminated. All cell lines were maintained in a humidified 37° C incubator with 5% CO_2_, and cell lines were tested and confirmed negative for mycoplasma, validated with MycoStrip (InvivoGen, San Diego, CA, USA, #rep-mys-50).

#### IRF Pathway Activation.

THP1 ISG Lucia cells were cultured according to manufacturer’s guidelines and used to measure the activation of the IRF Pathway. Briefly, cells were differentiated into macrophages using 150 nM Phorbol 12-myristate 13-acetate (PMA; Invivogen, San Diego, CA, USA) for 24 hrs. After differentiation, PMA-media was removed and replaced with basal growth media. After 72 hrs, the media was replaced with OPTIMEM (Gibco, New York, USA, #31985–070) prior to transfection. Cells were transfected with XFect Protein Transfection Reagent (TakaRa, Shiga, Japan, #631324). Cells were pre-treated, for 30 min with the 10 uM of caspase inhibitor, Q-VD-OPH (Sigma-Aldrich, Burlington, MA, USA, #SML0063), as indicated and transfected with either recombinant human Granzyme K (Sino Biological, #19732-H08H), recombinant human Granzyme B (Sino Biological, #10345-H08H) at indicated concentrations in OPTIMEM media for 3 hours. OPTIMEM was replaced with growth media and luciferin (a direct readout of IRF pathway) was collected from the cellular supernatant and measured at 24 and 48 hours post transfection using QUANTI-Luc: Luciferase Detection Reagent (Invivogen, San Diego, CA, USA, #rep-qlc4lg1).

#### IFN-α/β Pathway Activation.

IFNα/β Reporter HEK 293 cells were cultured according to the manufacturer’s guidelines to detect Type I interferons. Cells were transfected with XFect Protein Transfection Reagent (TakaRa, Shiga, Japan, #631324) with a caspase inhibitor, 10 uM Q-VD-OPH (Sigma-Aldrich, Burlington, MA, USA, #SML0063). Cells were transfected with either recombinant human Granzyme K (Sino Biological, #19732-H08H) or Granzyme B (Sino Biological, #10345-H08H) in OPTIMEM. STAT1/2-inducible secreted embryonic alkaline phosphatase (SEAP), was quantified utilizing the manufacturer’s guidelines at 24 and 48 hours transfection using QUANTI-Blue Solution (Invivogen, San Diego, CA, USA, #rep-qbs).

#### Immunofluorescence microscopy and analysis.

THP1 Macrophages and NS-SV-TTAC Acinar Cells were plated on an 8-well Lab-Tek II Chamber Slide (Thermo Scientific, 154534). After indicated treatment, cells were loaded with Quant-iT PicoGreen Reagent (1:50) (Invitrogen, #P7589A) and MitoTracker Red CMXRos (Invitrogen, #M7512) (1:10000) and incubated for 45 minutes at 37°C. The cells were fixed for 20 minutes at room temperature in 4% PFA. The cells were blocked in 3% BSA diluted in 1X PBS for 1 hour. Cells were washed with 1X PBS and then stained with primary antibodies overnight and then stained by secondary antibodies for 1 hour at room temperature (Supplemental Table 2). Images were acquired on Nikon A1 HD (Nikon) confocal microscope and processed with CellProfiler in ImageJ (Broad Institute).

#### Primary salivary gland epithelial cell (pSGEC) generation and culture.

pSGEC were generated according to methods published by Jang et al (*2015*)^63^. The pSGEC cell lines were not authenticated, although extensive characterization is published. Briefly, pSGECs were plated on collagen-coated 8-well chamber slides and transfected as indicated. Cells were washed with 1X PBS and immediately fixed with 4% PFA for 30 minutes at 37°C. The reaction was quenched via the addition of glycine (100 mM) for 20 minutes at room temperature. Cells were washed with 1X PBS and permeabilized with 1% TritonX-100 for 12 minutes at room temperature. The cells were blocked in 5% nonfat dry milk in PBS-T (blocking buffer) for 1 hour at room temperature. Cells were washed with PBS-T and then incubated with primary antibodies in blocking buffer and washed three times with PBS-T (10 min each) before incubated with the secondary antibodies (in blocking buffer) for 1 hour each at room temperature (Supplemental Table 2). After washing, cells were mounted with Fluoro-Gel II with DAPI (EMS) and images were acquired on an Olympus (FV1200 MP) confocal microscope and processed with CellProfiler in ImageJ.

#### AnnexinV Staining.

The potential for apoptosis induced by Granzyme K or B was measured using the flow-cytometry-based Dead Cell Apoptosis Kit with Annexin V Alexa Fluor 488 and Propidium Iodide (Invitrogen) following the manufacturer’s protocol on a FACS Symphony flow cytometer.

#### Western Immunoblot:

Extracted total protein was resolved using sodium dodecyl sulfate-polyacrylamide gel electrophoresis (SDS PAGE) (4% stacking, 12% resolving, Invitrogen) and transferred onto Polyvinylidene fluoride (PVDF) membranes. Membranes were incubated with primary and secondary antibodies (online Supplemental Methods Table 2). The signal was detected by ChemiDocMP Imaging System (BIO-RAD), and the density of the bands was analyzed using Fiji.

#### HiPlex RNAscope In Situ Hybridization.

12-plex RNAscope fluorescent in situ was performed on 5 μm-thick FFPE sections, using the RNAscope HiPlex12 Reagents Kit (Advanced Cell Diagnostics, Newark, CA, USA), according to the manufacturer’s protocols. The protocol was modified to incorporate antibody-based immunofluorescence. The following modifications were introduced: the slides were baked in a dry air oven for 30 min at 60°C. After rehydration the slides were processed for target retrieval using 1X Co-Detection Target Retrieval, during 20 min. The slides were quickly rinsed in water and then washed in PBS-T (PBS 1X, 0.1 % Tween-20, pH 7.2). A hydrophobic barrier was created using ImmEdge pen and primaries antibodies were added to detect pCTK (Novus Bio, NBP2–29429, dilution 1:200 in Co-detection Antibody Diluent from ACD), ACTA2 (Antibodies.com, #AB2445, dilution 1:300 in Co-detection Antibody Diluent from ACD) and CD45 proteins (NB100–77417SS, dilution 1:300 in Co-detection Antibody Diluent from ACD) and incubated at 4°C overnight. The next day primary antibodies were washed in PBS-T, three times for 2 min at room temperature. Tissues and primary antibodies were post-fixed in 10% Neutral Buffered Formalin (NBF) for 30 min at room temperature and washed in PBS-T thrice for 2 min. Tissues were then digested using RNAscope Protease III during 30 min at 40°C. Immediately continuing with the HiPlex assay according with standard protocol. The following RNAscope HiPlex probes were used: Hs-CST3(T1), Hs-PIP(T2), Hs-WFDC2 (T3), Hs-BPIFB2 (T4), Hs-CFTR(T5), Hs-B2M(T6), Hs-ZG16B(T7), Hs-ISG15(T8), Hs-PRR4(T9), Hs-AMY1A(T10), Hs-MUC7(T11), Hs-MUC5B(T12). All tissues were first run with positive and negative control probes (species-specific) to ensure tissue and RNA quality for RNAscope ISH.

#### Recursive Image Acquisition.

After each round of HiPlex RNAscope hybridization, fluorescent images were acquired on Axio Scan-Z1 (Zeiss) fluorescent slide scanner. Whole slide images were collected with a 40x/0.95 objective, with a 0.5 μm step size. Images for each individual in the study were collected from at least 2–4 salivary glands. After each round of imaging for HiPlex RNAscope, coverslips were removed by submerging the slides in SSC 5X buffer until coverslips fall off the slide. Subsequently, fluorophores were cleaved, autofluorescence quenched, and then new fluorophores were added (Advanced Cell Diagnostics, Newark, CA, USA). Immediately after imaging was repeated on the same tissue slices using the additional ISH probes. We performed this for three rounds of RNAscope, after final ISH imaging step, fluorophores were cleaved, autofluorescence quenched and the following secondary antibodies were added: donkey-antiMouseAF488, donkey-antiRabbitAF446 and donkey-antiRat647, Jackson Immunoresearch, all in dilution 1:300 in Co-detection Antibody Diluent from ACD. Finally, antibodies were washed in PBS-T and mounted in prolong-Diamond antifade with DAPI. A final round of imaging was performed to detect the signal of the secondary antibodies.

#### Image Processing: Registration, Segmentation, Quantification of RNAscope Puncta.

After collecting the images, the following process was used to register the individual fluorescent images: First the set of whole slide images to analyze was defined as a QuPath project. The project was then opened in Fiji, and the registration was performed using both elastix^64^, and a semi-automated approach using BigWarp to interactively improve the results of the automated registration. The transformations were retrieved and applied in QuPath to produce on-the-fly transformed images that correspond to superimposition of all four rounds of images per slide. Once the overlayed images were produced in QuPath, new OME-tiff files were exported, the new 19-channel images were used to create a new Qupath project. Cells were segmented using CellPose developing our own model^65,66^. Once the cells were segmented, we quantify the number of dots per cell using the subcellular detection in QuPath.

#### HiPlex ISH analysis.

The matrix of counts of dots per cell obtained from Qupath was cleaned and parsed in R. *MUC7* mRNA positive cells were subset to identify SMACs subtypes. Log2-transformed gene expression of *WFDC2*, *CST3, PRR4, ZG16B, MUC7*, and *MUC5B* were used as inputs UMAPS were obtained using the uwot package in R, the clusters were classified based on the expression of *WFDC2, CST3, PRR4, ZG16B, MUC7,* and *MUC5B*, and their similarity to reference cell types identified in scRNAseq. With this set of cell type identification, we ran a co-occurrence test using SPIAT^67^. After reclassification, cell type proportions were calculated for each of the patients in the study and the cell proportions were compared between nonSjD and SjD using a mixed model logistic regression on the package lme4^68^.

#### General statistical methods.

All non-high throughput data (e.g., analysis of biological assays) were analyzed using Prism 9. Appropriate statistical tests were selected based on data type and are described throughout the text. Figures were constructed using Prism 9 when not otherwise described elsewhere.

## Supplementary Material

Supplement 1

## Data Availability

10X Single Cell and Visium spatial transcriptomic data has been uploaded to CZ CELLxGENE Discover (www.https://cellxgene.cziscience.com) and will be available at https://cellxgene.cziscience.com/collections/21bbfaec-6958-46bc-b1cd-1535752f6304. Spatial phenotyping (PhenoCycler-Fusion) and spatial transcriptomic (HiPlex ISH) data are available at Mendeley Data: DOI: 10.17632/b5j9dhgyp4.1 https://data.mendeley.com/preview/b5j9dhgyp4?a=3eb748ca-8f09-4e2b-8b09-2281350a5c94. Code used for the analyses is available at the following websites:https://github.com/pranzatelli/scRNAseqNM2023, https://github.com/huynhkl953/Sjogren_paper, and CellProfiler pipelines are available at https://cellprofiler.org/published-pipelines. All other data is available upon request.
