# Supplementary material for: GZMK+CD8+ T cells Target A Specific Acinar Cell Type in Sjögren’s Disease
Source: Res Sq. 2024 Jul 11:rs.3.rs-3601404. Originally published 2023 Dec 19. Preprint. [Version 2] doi: 10.21203/rs.3.rs-3601404/v2 (PMC10775371; doi:10.21203/rs.3.rs-3601404/v2)
Supplement: Supplement 1 [file NIHPPrs3601404v2-supplement-1.pdf]

Supplemental Figures:

Figure S1. (a) Barplot of changes in cell proportion in disease and SSA positivity across all cell types. (b) Dotplot of genes found to be specific to Leiden clusters and used to annotate those clusters. (c) Dotplots of differentially expressed genes in Sjögren's and the cell types those genes are differentially expressed in. (d) Dotplots of differentially expressed genes in SSA+/- and the cell types those genes are differentially expressed in. (e) Dotplots of genes differentially expressed across Sjögren's and anti-SSA positivity and the cell types those genes are differentially expressed in.

Figure S2. (a) Distribution of marker genes in SjD and non-SjD ISH samples. Samples are downsampled so that an equal number of cells are taken from all samples. (b) UMAP of the cells from ISH. There is a clear division by patient diagnosis. (c) UMAP colored by ISH quantification.

Figure S3. (a) UMAP colored by Gaussian mixture model (GMM) cluster. The sample number of clusters were used as input channels from the ISH data. (b) Distribution of ISH quantification for each cluster of cells. (c) Samples with cells colored by GMM cluster. GMM clusters broadly correspond to acinar, interstitial or immune tissue from one of three groups of patients: patients with a high focus score, patients with a low focus score or no focus score and antibody positivity, or patients who were antibody negative and had no focus score. Two non-SjD patients were outliers and clustered uniquely.

Figure S4. Transforming scRNA-seq data from gene expression to indices (a) reveals new organizations of cells and clinical feature-specific clusters. (b) UMAP of cells after transformation to an index S-score space with coloring by a new application of the Leiden clustering algorithm. (c) Proportions of index clusters that represent annotations in the original single-cell space. Some clusters are close to a single cell type; others contain a variety of cell types. There are no one-to-one mappings between index clusters and original annotations. (d) UMAP on index S-scores colored by original annotation. Some clusters are enriched for clinical features and multiple regions are homogenous in their clinical phenotype. (e) The original scRNA-seq UMAP overlaid with the index clusters. The two most specific index clusters are both found in the T cell island in the UMAP as well as the clusters associated with antigen-presenting cells. (f) Ratios of Sjögren's to non-Sjögren's and anti-SSA positive to negative patients in each of the index clusters. Two index clusters are found at the extremes of the plot: one with high SjD specificity and the other with high anti-SSA positive specificity. These index clusters are composed of T cells, B cells, dendritic cells, and macrophages.

Figure S5. Interferon response increased across cell types with SSA positivity. (a) Cells in the index UMAP colored by patient’s diagnosis and SSA positivity. (b) Changes in index expression with disease diagnosis and SSA positivity. (c) Feature plots of IFN score in SSA+ and SSA- patients. Except in M2 macrophages and endothelial populations, IFN response score is low across cell types in SSA negative individuals. (d) Barplot of IFN response S-score across cell types in SSA positive and negative patients. IFN response increases in every cell population.

Figure S6. PhenoCycler-Fusion 35-plex spatial proteomics confirms changes in glandular composition and cellular interactions. (a) Dot plot of multiple protein expression of cellular phenotypes (left-side of dot plot; EPCAM to CD20) and cellular state markers (right side of dot plot; HHP3 to IFNG). (b) Cellular proportion changes in SjD compared with nonSjD. (c) Per-cell log2 fold-changes in protein expression in SjD compared with non-SjD were calculated for all phenotype and segmented cells. (d) Cellular neighborhoods in SjD compared with non-SjD. (e) CellChat ligand-receptor analysis corroborating neighborhood analysis and the importance of B- and T-lymphocyte co-stimulatory signaling in SjD. (f) Representative PhenoCycler-Fusion immunofluorescent images from a lymphocytic focus showing infiltration with T- and B-lymphocytes around ducts and acini.

Figure S7. (a) Evaluation of the induction of apoptosis after transfection of recombinant GZMK, GZMB, β-Gal, or Naive (mock) in immortalized NS-SV-TTAC Acinar Cells using Annexin V/PI staining and flow cytometric analysis. 100,000 events were measured and performed in biological duplicate (except for β-Gal) *- $p<0.05$, ANOVA. (b) Optimization of protein transfection with GZMK in HSG cells. Granzyme K levels were normalized to β-Actin and determined ratios are compared to control. (c) Transfection of GZMK and GZMB into NS-SV-TTAC exhibits time-dependent degradation over 48 hours. Granzyme K and B levels were normalized to β-Actin and determined ratios are compared to naive Sample. (d) GZMK transfection induced dose-dependent increases in pIRF3 by Western immunoblot in pSGEC. (e) THP1-macrophages were transfected with proteins (i.e., GZMK, GZMB, GFP, and mock) and analyzed by fluorescence microscopy showing GZMK induces cytosolic relocalization of mtDNA to the cytosol. Image is representative of the findings from three biological replicates. (f) Engineered cell lines were used to measure IRF Pathway and Type I IFN induction using THP1-ISH Lucia and HEK-IFNa/b SEAP cells, respectively. Assays were performed on technical triplicates from biological duplicates and compared using ANOVA; results presented are a single representative experiment from the three experimental replicates.

Figure S8. (a) Representative marker genes spatial plots for seromucous (*MUC7*) and mucous (*MUC5B*) acini, fibroblasts (*LUM*), and T cells (*CD3E*). Note SjD accompanies major shifts in seromucous acinar cell (SMAC) cell composition, T cells, and fibrosis. (b) Cartoon depicting cellular colocalization and cooccurrence analyses. Reference cell types were integrated from existing scRNA-seq (Figure 1e) data using Cell2Location to infer cell type locations in spRNA-seq. (c) Autocorrelations of cell types form a colocalization matrix that reconstructs the usual architecture of the glands. In SjD, the general architecture remains preserved; however, shifts in colocalizations can be appreciated in multiple cell types. (d) Shown are CD8+ exhausted T cells and P*RR4+CST3+WFDC2*- seromucous acinar cells (SMACs) along with manually annotated ducts and immune infiltrates. Top tissue is from a healthy volunteer and bottom tissue is from a patient with a diagnosis for Sjögren's.

Supplemental Methods Table 1: List of antibodies and dyes used for flow cytometry of minor salivary glands.

Antigen	Species	clone	conjugate	Manufacturer	Cat#
CD3	mouse	OKT3	BV785	BioLegend	317330
CD4	mouse	SK3	BUV737	BD	612748
CD8	mouse	RPA-T8	BUV496	BD	564804
CD19	mouse	HIB19	AlexaFluor700	BioLegend	302226

CD45	mouse	HI30	PE	BioLegend	304058
Fixable Viability Dye eFluor 780	N/A	N/A	N/A	eBioscience	65-0865-18
IFNg	mouse	B27	BUV395	BD	563563
TNFa	mouse	MAb11	BV421	BioLegend	502932
IL-17A	mouse	eBio64DEC17	PECy7	eBioscience	25-7179-42
CD107a	mouse	H4A3	BV605	BioLegend	328634

Supplemental Methods Table 2. List of PhenoCycler-Fusion Validated Antibodies used for 35-plex Spatial Phenotyping

PCF Antibody	Clone	Barcode/Reporter	Wavelength
CD20	L26	BX/RX020	AF750
CD8A	C8/144B	BX/RX026	Atto550
CD4	EPR6855	BX/RX003	AF647
GZMB	D6E9W	BX/RX041	Atto550
FOXP3	236A/E7	BX/RX031	AF647
EPCAM	D9SEP	BX/RX091	AF750
Ki67	B56	BX/RX047	Atto550
PHH3	AKYP0060	BX/RX030	AF647
HLA-A	EP1395Y	BX/RX004	AF750
Galectin-3	M3/38	BX/RX035	Atto550
CD3E	EP449E	BX/RX045	AF647
CD45RO	UCHL1	BX/RX017	Atto550
CD45	D9M81	BX/RX021	AF647
CD21	AKYP0061	BX/RX032	Atto550
PD-L1	73-10	BX/RX043	AF647
CD14	EPR3653	BX/RX037	Atto550
PD-1	D4W2J	BX/RX046	AF647
MPO	AKYP0113	BX/RX098	Atto550
CD68	KP1	BX/RX015	AF647
GATA3	D13C9	BX/RX049	Atto550
IDO1	V1NC3IDO	BX/RX027	AF647
CD31	EP3095	BX/RX001	AF750
KRT14	Poly19053	BX/RX002	Atto550
CD107a	H4A3	BX/RX006	AF647
KRT8/18	C51	BX/RX081	AF750
CD141	AKYP0124	BX/RX087	Atto550
ICOS	D1K2T	BX/RX054	AF647
SMA	AKYP0081	BX/RX013	AF750
PDPN	NC-08	BX/RX023	Atto550
COL_IV	EPR20966	BX/RX042	AF647
CD34	AKYP0088	BX/RX025	Atto550
HLA-DR	EPR3692	BX/RX033	AF647
CD38	E7Z8C	BX/RX089	Atto550
Bcl2	EPR17509	BX/RX085	AF647
VIM	O91D3	BX/RX022	AF750
IFNG	AKYP0074	BX/RX020	Atto550
CD66A/C/E	ASL-32	BX/RX016	AF647
PanCK	AE-1/AE-3	BX/RX019	AF750
CD56	CAL53	BX/RX028	Atto550
CD11c	118/A5	BX/RX024	AF647

Supplemental Methods Table 3: Reagents and Dilutions

**Reagents**	**Manufacturer (Catalog Number)**	**Dilution**	**Lot Number**	**Clone Name**
**Immunofluorescence Confocal Microscopy**				
Quant-iT PicoGreen Reagent	Invitrogen (P7589A)	1:50	2600152	–
MitoTracker Red CMXRos	Invitrogen (#M7512)	1:10000	2575951	—
6-His Tag Antibody (A190-113A) Goat Polyclonal	Bethyl	1:200		Polyclonal
Phospho-IRF-3 (Ser396) (D601M) Rabbit mAb	Cell Signaling Technology (#29047S)	1:100	5	
Granzyme K Antibody (PA5-50980) Rabbit Polyclonal	Invitrogen	1:100	YE3917452	Polyclonal

**Western Blots**				
HRP-conjugated anti β-actin Mouse mAb	Sigma-Aldrich (#A3854)	1:10000	100M4782	AC-15
Phospho-IRF-3 (Ser396) (D601M) Rabbit mAb	Cell Signaling Technology (#29047S)	1:300	5	
IRF-3 (D6I4C) Rabbit mAb	Cell Signaling Technology (#11904S)	1:300	7	
Granzyme K Antibody (PA5-50980) Rabbit Polyclonal	Invitrogen	1:300	YE3917452	Polyclonal
Purified Mouse Anti-Granzyme B	BD Biosciences (#550558)	1:300	1288810	
RDye® 800CW Donkey anti Rabbit IgG (H + L)	LI-COR Biosciences (#926-32213)	1:1000 000	D20503-13	—
IRDye® 680RD Donkey anti-Mouse IgG (H L)	LI-COR Biosciences (#926-68072)	1:10000	C81107-05	—
SuperSignalTM West Pico PLUS Chemiluminescent Substrate	Thermo Scientific (#34580)	-	XF348581	—

Supplemental Methods Table 4: Summary of packages and software used in the acquisition and analysis of Hiplex transcriptomics data.

Packages (version)	Availability	Reference
Uwot (0.1.16)	https://github.com/jlmelville/uwot	Melville J (2023). _uwot: The Uniform Manifold Approximation and Projection (UMAP) Method for Dimensionality Reduction.
Zen Blue software (3.1)	Comercial from Zeiss	
0.4	https://github.com/qupath/qupath/releases/tag/v0.4.4	Bankhead, P. et al. QuPath: Open source software for digital pathology image analysis. Scientific Reports (2017).
	https://github.com/SuperElastix/elastix	S. Klein, M. Staring, K. Murphy, M.A. Viergever, J.P.W. Pluim, "elastix: a toolbox for intensity based medical image registration," IEEE Transactions on Medical Imaging, vol. 29, no. 1, pp. 196 - 205, January 2010. download doi<br><br>D.P. Shamonin, E.E. Bron, B.P.F. Lelieveldt, M. Smits, S. Klein and M. Staring, "Fast Parallel Image Registration on CPU and GPU for Diagnostic Classification of Alzheimer's Disease", Frontiers in Neuroinformatics, vol. 7, no. 50, pp. 1-15, January 2014. download doi
	https://imagej.net/software/fiji/downloads	Schindelin, J., Arganda-Carreras, I., Frise, E., Kaynig, V., Longair, M., Pietzsch, T., … Cardona, A. (2012). Fiji: an open-source platform for biological-image analysis. *Nature Methods*, *9*(7), 676–682. doi:10.1038/nmeth.2019
	https://forum.image.sc/t/warpy-registration-of-whole-slide-images-at-cellular-resolution-with-fiji-and-qupath/61803	Nicolas Chiaruttini, Olivier Burri, Peter Haub, Romain Guiet, Jessica Sordet-Dessimoz, Arne Seitz.<br>An Open-Source Whole Slide Image Registration Workflow at Cellular Precision Using Fiji, QuPath and Elastix. Frontiers Comput. Sci. 3: 780026 (2021)
e	https://github.com/BIOP/qupath-extension-cellpose	Stringer, C., Wang, T., Michaelos, M. et al Cellpose: a generalist algorithm for cellular segmentation. Nat Methods 18, 100–106 (2021).<br>Pachitariu, M., Stringer, C. Cellpose 2.0: how to train your own model. Nat Methods 19, 1634–1641 (2022).<br>Cutler, K.J., Stringer, C., Lo, T.W. et al. Omnipose: a high-precision morphology-independent solution for bacterial cell segmentation. Nat Methods 19, 1438–1448 (2022).
	https://cancer-evolution.github.io/SPIAT/articles/introduction.html	Feng, Y., Yang, T., Zhu, J. *et al.* Spatial analysis with SPIAT and spaSim to characterize and simulate tissue microenvironments. *Nat Commun* 14, 2697 (2023).
	https://github.com/lme4/lme4	Bates D, Mächler M, Bolker B, Walker S (2015). "Fitting Linear Mixed-Effects Models Using lme4." *Journal of Statistical Software*, **67**(1), 1–48
