# Supplementary material for: GZMK+CD8+ T cells Target a Specific Acinar Cell Type in Sjögren’s Disease
Source: Res Sq. 2023 Dec 19:rs.3.rs-3601404. Preprint. [Version 1] doi: 10.21203/rs.3.rs-3601404/v1 (PMC10775371; doi:10.21203/rs.3.rs-3601404/v1)
Supplement: Supplementary file 1 [file NIHPPRS3601404V1-supplement-1.pdf]

Supplemental Table 2: Reagents and Dilutions

Reagents		Manufacturer	Dilution
**Immunofluorescence Confocal Microscopy**			
Figure 5	Quant-iT PicoGreen Reagent	Invitrogen	1:50
Figure 5	MitoTracker Red CMXRos	Invitrogen	1:10000
Figure 5	6-His Tag Antibody (A190-113A) Goat Polyclonal	Bethyl	1:200
Figure 5	Phospho-IRF-3 (Ser396) (4D4G) Rabbit mAb	Cell Signaling Technology	1:100
Figure 5	Granzyme K Antibody (PA5-50980) Rabbit Polyclonal	Invitrogen	1:100
**Western Blots**			
HRP-conjugated anti β-actin Mouse mAb		Invitrogen	1:10000
Phospho-IRF-3 (Ser396) (4D4G) Rabbit mAb		Cell Signaling Technology	1:300
IRF-3 (D83B9) Rabbit mAb		Cell Signaling Technology	1:300
Granzyme K Antibody (PA5-50980) Rabbit Polyclonal		Invitrogen	1:300
Granzyme B Antibody (MA1-80734) Mouse mAb		Invitrogen	1:300
RDye® 800CW Donkey anti Rabbit IgG (H + L)		LI-COR Biosciences	1:10000
IRDye® 680RD Donkey anti-Mouse IgG (H + L)		LI-COR Biosciences	1:10000
SuperSignalTM West Pico PLUS Chemiluminescent Substrate		Thermo Scientific	-

HiPlex RNAscope In Situ Hybridization.
